# A comparison of EFECE systems with tension band wiring for patella fracture fixation in cadavers

**DOI:** 10.1186/s13018-020-01781-9

**Published:** 2020-07-10

**Authors:** Emre Karadeniz, Elif Nedret Keskinoz

**Affiliations:** 1 Orthopedics and Traumatology Department, School of Medicine, Kocaeli University Hospital, İzmit, Turkey; 2 Anatomy Department, School of Medicine, Acıbadem Mehmet Ali Aydınlar University, Istanbul, Turkey

**Keywords:** Osteosynthesis, Patellar fracture, Implant, Fracture fixation, Internal fixator

## Abstract

**Background:**

EFECE systems are newly defined internal fixation systems, which are suitable for patella fracture fixation. The aim of this study was to compare the fixation strength of EFECE Systems with tension band wiring for transverse patellar fracture simulation on fresh frozen cadaver models.

**Methods:**

Quadriceps tendon-patella-patellar tendon (QT-P-PT) complex was prepared from human cadavers. After simulation of a transverse patella fracture, in group 1, 5 patella were fixed with a pair of 1.2 mm EFECE wires and 4 EFECE devices. In group 2, 5 patella were fixed with a pair of 1.2 mm Kirschner wires (K-wire) and a cerclage wire according to the tension band technique.

Using a testing device with custom-made jaws, increasing distraction force was applied to these QT-P-PT complexes. Extension of these complexes with the distraction forces was observed. The maximum distraction force and the elongation during maximum force were evaluated.

**Results:**

After 5 experiments with the EFECE systems, there was no EFECE wire breakage or EFECE wire-EFECE device catching failure. The median maximum force was 740 N (720-810 N). During maximum distraction force the median extension was 2.5 mm (1.6-2.5 mm). After 5 experiments with the tension band technique, there was no K-wire breakage. The median maximum force was 330 N (240-510 N). During this maximum distraction force the median extension was 3.4 mm (2.2-3.8 mm).

**Conclusions:**

Based on the biomechanical advantages, patella fracture treatment with EFECE systems may constitute a reasonable alternative in the treatment of patella fractures.

## Introduction

The patella is the largest sesamoid bone in the human body and fractures of this bone account for 1% of all skeletal system fractures [1]. Conservative treatment is indicated to fractures with minimal displacement and those with no articular surface incongruency. If fragment displacement is more than 2 mm or there is articular incongruency, surgical treatment is indicated [2]. For the surgical treatment of patella, several techniques and implant materials have been described [3].

The tension band technique, defined by Müller et al. in 1979, became the standard technique for patella fracture fixation [4]. In this open fixation technique, 2 parallel Kirschner wires (K-wire) and a cerclage wire compress the fracture line and cause compression of the articular surface with distraction forces.

EFECE systems are newly defined and patented, internal fixation implants, which also have the indication scale for patella fractures. EFECE systems comprise an EFECE device, EFECE wire and surgical tools. The EFECE device functions as a screw head on the EFECE wire to compress and fix the fracture line.

The aim of this study was to compare the conventional tension band technique with the new EFECE systems to determine whether the new EFECE systems could resist greater distraction force and cause less displacement during maximum force.

## Material and method

**Fig. 1:**
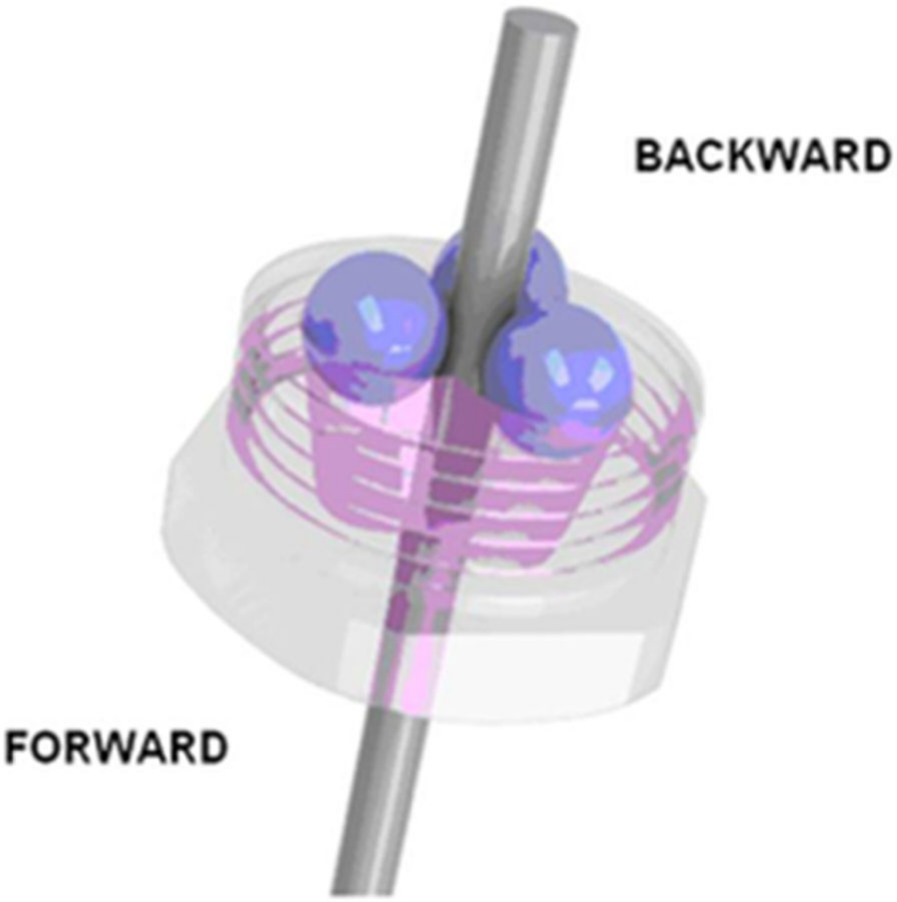
The EFECE implant locking mechanism is applied with the help of balls in the cone-shaped gloves. In forward movement to compress the fractured fragments across the K-wire, the balls move back to the base of the cone and release the K-wire. When pulled back, the balls move to the narrow part of the cone and lock the K-wire

EFECE systems comprise an EFECE device, EFECE wire and surgical tools. The EFECE device (EFECE, Akım Metal, 2015, Istanbul, Turkey) is cylinder shaped with a 6-mm radius and a 5-mm length that features a hole for the insertion of a 1.2-mm EFECE wire (EFECE, Akım Metal, 2015, Istanbul, Turkey). The EFECE device contains 2 threaded pieces that connect to each other. The top piece functions as a cap, whereas the second piece contains 3 gloves for the insertion of 3 balls. These balls are 1.5 mm in diameter. The locking mechanism is aided by the balls in the cone-shaped gloves. In forward movements, the balls move back to the base of the cone, and during pulling movements, the balls move to the narrow part of the cone and lock the EFECE wire [5] (Fig. 1). The surgical kit for EFECE systems (EFECE, Akım Metal, 2015, Istanbul, Turkey) is for compression of the fracture line, locking and unlocking the EFECE device.

With the approval from our institution’s institutional review board (KUGOKAEK) numbered 2020/89, a total of 11 fresh cadavers (7 female and 4 male; age range, 55-94 years; mean age, 74.5 years) which were acquired from the Acıbadem Mehmet Ali Aydinlar University Anatomy Department were used in this study. None of the knees had undergone surgery or trauma to the extensor mechanism. The dissections were performed in Acıbadem Mehmet Ali Aydinlar University Atasehir, Istanbul, Turkey. The study was conducted according to the guiding principles of the Declaration of Helsinki.

A quadriceps tendon-patella-patellar tendon (QT-P-PT) complex was prepared from each cadaver. Transverse patellae fractures were created in the same localization in all the samples. In group 1, 5 patella were fixed with a pair of 1.2 mm EFECE wires and 4 EFECE devices. The EFECE devices were driven mutually on EFECE wires until contact was made with the bone cortex c. Then, these devices were compressed and tightened using the surgical tools.

In group 2, 5 patella were fixed with a pair of 1.2 mm K-wires and a cerclage wire according to the tension band technique.

**Fig. 2:**
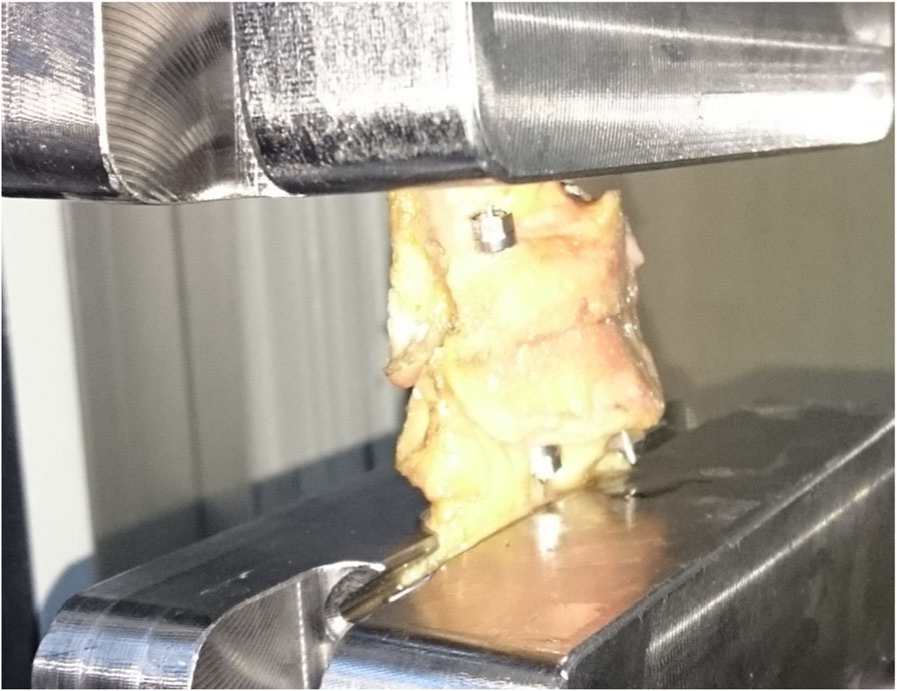
QT-P-PT complexes fixed with EFECE systems or tension band wiring were attached to the jaws of the material testing system servo hydraulic testing machine and increasing distraction force was applied to these systems

**Fig. 3:**
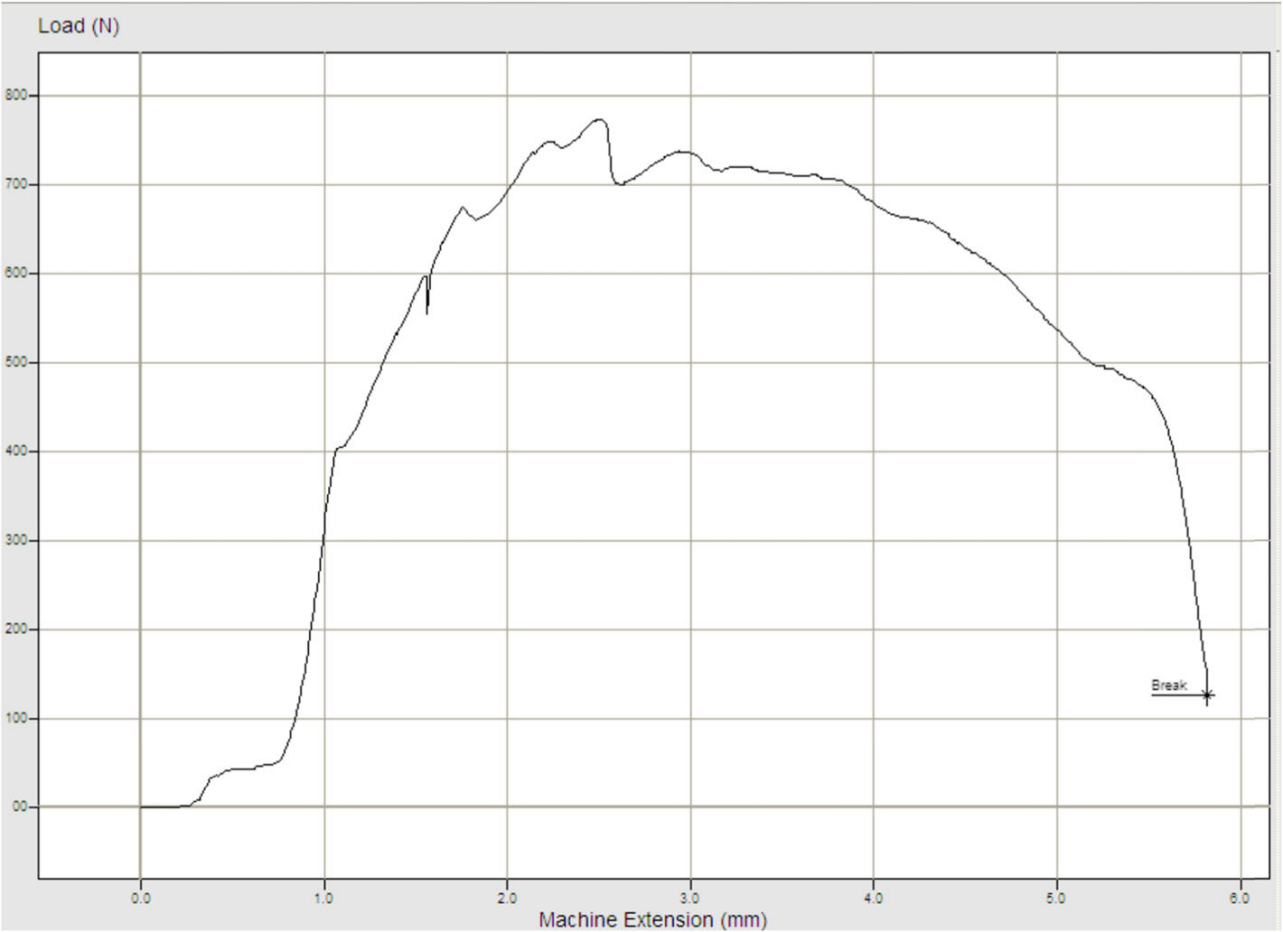
Evaluation of the maximum distraction force and the total extension during maximum force

Using a servo hydraulic testing machine with custom-made jaws (Instron Inc, Norwood, Massachusetts, USA), increasing distraction force (1 cm/min) was applied to these QT-P-PT complexes (Fig. 2). The maximum loads and the elongations during maximum load were observed and recorded with the Nexygen software (Fig. 3).

All statistical analyses were performed using IBM SPSS for Windows version 20.0 (IBM Corp., Armonk, NY, USA). The Kolmogorov-Smirnov test was applied to test the normality of data distribution. Continuous variables were expressed as median values (25th-75th percentiles). Comparisons of continuous variables between the groups were performed using the Mann-Whitney *U* test. A two-sided value of *p* < 0.05 was considered statistically significant.

## Results

After 5 experiments with EFECE system fixation, the median maximum load was 740 N (720-810 N). During these maximum loads, the median extension was 2.5 mm (1.6-2.5 mm).

After 5 experiments with tension band wiring, the median maximum load was 330 N (240-510 N). During these maximum loads, the median extension was 3.4 mm (2.2-3.8 mm).

The maximum load level of the EFECE system fixation was determined to be more than 2 times greater than that of tension band wiring (Table 1, *P =* 0.008). No statistically significant difference was determined between the EFECE system and tension band wiring in respect of extension (Table 1, *P =* 0.095).

**Table 1:** Maximum load resistance of the fixation and the extension with that maximum load

	EFECE (*n* = 5)	Tension band wiring (*n* = 5)	*P*
	Median (25th-75th per.)	Median (25th-75th per.)	
Load (N)	740.00 (720.00-790.00)	330.00 (265.00-425.00)	0.008
Extension (mm)	2.50 (2.00-2.50)	3.40 (2.60-3.75)	0.095

## Discussion

Selecting the best surgical approach, appropriate method and materials for treating patellar fractures is a tough challenge for orthopedists [6–11]. The main goal with surgical treatment of patella fractures is to provide a congruent articular surface and maintain rigid fixation. Open reduction and tension band wiring technique widely accepted by the orthopaedic trauma surgeons because of mostly achieving these goals [12, 13].

Although open reduction and internal fixation techniques are the standard method for patellar fracture surgical treatment, there are many reports describing disadvantages and complications associated with traditional surgical treatments [10, 14]. Technique modifications such as cannulated screws, cannulated screws with tension band wire and plate fixation have been defined to enhance the surgical outcome [15–18]. And also for biomechanical comparison of these techniques, scoring methodology has been developed to assess the quality of biomechanical studies [19].

LeBrun et al. evaluated functional outcomes after surgically treatment of patella fractures [20]. In this study, with evaluation of validated outcome measures as well as objective physical findings it was concluded that functional deficits and significant symptomatic complaints persist after patella fracture operative treatment. These results have encouraged scientists to define “new and minimally invasive” implant technologies.

With the patented EFECE systems, fractures can be treated with an internal fixation technique. EFECE systems include an EFECE device, EFECE wire and surgical kit. The EFECE device is cylinder shaped with a hole for the insertion of the EFECE wire. The surgical kit for EFECE systems comprises a sleeve, working cannula, screwdrivers, wire tensioner, wire cutter and magnet. The surgical kit is designed for use in the percutaneous surgical technique. After reduction of the fracture and EFECE wire placement as in the conventional technique, using the sleeve, working cannula and screwdrivers, two reciprocal EFECE devices are pushed forward on EFECE wires until contact is made with the bone cortex. At this stage, there is no need to rotate the screwdriver on the EFECE wire and there is no need to measure the diameter of the implants, which are time-consuming procedures for screw insertion. Compression force can then be applied on the fracture fragments with adjustment of the EFECE wire tension using the wire tensioner. Once the implants are locked with the screwdrivers, these devices do not allow forward or backward movement on the EFECE wires. The remaining part of the wire should be cut with the percutaneous wire cutter.

As the balls in the locking mechanism are magnetically active, these implants can be removed with magnets. This implant removal technique is also an easy and new approach in orthopaedic surgery.

There are limitations of the EFECE systems. The EFECE system needs at least two reciprocal EFECE devices for wire fixation. The counter part of the EFECE wire that goes through the fragments and leaves the bone cortex needs to be prepared for EFECE insertion. The fixation strength of the EFECE system is dependent on the mechanical properties of a thin EFECE wire.

The findings of this study suggest that osteosynthesis of patella fractures with EFECE systems provides sufficient mechanical stability to prevent fracture separation with postoperative functional loading. Compared with tension band wiring, the EFECE systems demonstrated greater fixation strength and no difference in fracture gap distraction. The tension band wiring failed at significantly lower loads than the EFECE systems (*P =* 0.008).

In this study, the failure mechanism with EFECE systems was seen to be due to failure of the bone with sinking of the EFECE device into the patella. There were no failures in the EFECE device–EFECE wire interspace. The failure mechanism with tension band wiring was due to slippage of the cerclage wire and then sinking of the K-wire into the patella.

## Conclusion

Patella fracture fixation with EFECE systems provides a biomechanical stable fixation. Compared with tension band wiring, EFECE systems fixation resulted in higher mechanical strength and reduced fragment dislocation under loading. In comminuted patella fractures, more than 2 EFECE systems in different alignments can be used to compress and fix the fractured fragments with the percutaneous technique. Based on these biomechanical findings, EFECE systems may constitute a reasonable alternative to tension band wiring in the treatment of patella fractures.

## Data Availability

All data generated or analysed during this study are included in this published article.

## Abbreviations

QT-P-PT: Quadriceps tendon-patella-patellar tendon
K-Wire: Kirschner wire
N: Newton
